# Archaeal Ubiquitin-Like Proteins: Functional Versatility and Putative Ancestral Involvement in tRNA Modification Revealed by Comparative Genomic Analysis

**DOI:** 10.1155/2010/710303

**Published:** 2010-09-26

**Authors:** Kira S. Makarova, Eugene V. Koonin

**Affiliations:** National Center for Biotechnology Information, NLM, National Institutes of Health, Bethesda, MD 20894, USA

## Abstract

The recent discovery of protein modification by SAMPs, ubiquitin-like (Ubl) proteins from the archaeon *Haloferax volcanii*, prompted a comprehensive comparative-genomic analysis of archaeal Ubl protein genes and the genes for enzymes thought to be functionally associated with Ubl proteins. This analysis showed that most archaea encode members of two major groups of Ubl proteins with the *β*-grasp fold, the ThiS and MoaD families, and indicated that the ThiS family genes are rarely linked to genes for thiamine or Mo/W cofactor metabolism enzymes but instead are most often associated with genes for enzymes of tRNA modification. Therefore it is hypothesized that the ancestral function of the archaeal Ubl proteins is sulfur insertion into modified nucleotides in tRNAs, an activity analogous to that of the URM1 protein in eukaryotes. Together with additional, previously described genomic associations, these findings indicate that systems for protein quality control operating at different levels, including tRNA modification that controls translation fidelity, protein ubiquitination that regulates protein degradation, and, possibly, mRNA degradation by the exosome, are functionally and evolutionarily linked.

## 1. Introduction

Ubiquitination (ubiquitylation) of proteins is an ancestral, pivotal process in eukaryotes that governs protein trafficking and turnover, signaling, heterochromatin remodeling, and other processes [1–3]. All eukaryotes possess an elaborate system that includes a variety of small proteins of the ubiquitin (Ub) family, E1 Ub-activating, E2 Ub-conjugating, and E3 Ub-ligase enzymes, as well as a broad diversity of deubiquitinating enzymes (DUBs) [1, 2, 4]. Ubiquitin conjugation through the formation of isopeptide bonds by the e-amino groups of two conserved lysines of the Ub molecule (K48 and K63) determines the fate of most proteins in eukaryotic cells, in terms of both topogenesis and degradation. The functioning of Ub-centered signaling systems is regulated through the activities of numerous, specific Ub-binding domains and proteins. 

Ubiquitin is one of the most highly conserved eukaryotic proteins, and the evolution of the Ub system is fairly well studied [1, 5–8]. In particular, it has been shown that Ub homologs in bacteria and most likely in archaea are involved in thiamine and molybdenum (Mo)/tungsten (W) cofactor biosynthesis along with functionally linked homologs of E1 enzymes; in addition, E2 family proteins and homologs of metal-dependent DUBs of the Jab1/MPN family have been detected in several bacteria in association with Ub-like (Ubl) and E1-like proteins, leading to the hypothesis that these proteins could give rise to the Ub-system of eukaryotes; in contrast, E3 enzymes appear to be specific to eukaryotes [1, 7]. Indeed, there are some steps of thiamine and Mo/W cofactor biosynthesis that are biochemically equivalent to Ub conjugation. These steps include incorporation of sulfur into the respective molecules mediated by the Ubl sulfur-carrier proteins of the ThiS or MoaD family. These Ubl proteins are activated by adenylating E1-like enzymes of the ThiF and MoeB families, and in the next step, sulfur is incorporated by sulfur transferases of the IscS or rhodanese family, that transfer sulfur to its target via an intermediate persulfide (-S-S-H) formed by the active site cysteine [1, 7, 9–13].

The eukaryote Ub proteins and the prokaryote ThiS/MoaD family proteins possess the same *β*-grasp fold [14, 15] and a conserved carboxyl-terminal glycine which is crucial for the activation by E1-like enzymes [9, 10, 12, 13]. Recently, a protein modification system, known as pupylation, that is functionally equivalent but not homologous to the Ub system has been discovered in *Mycobacterium tuberculosis* [16, 17]. The two key components of this system are the small protein Pup and the enzyme PafA that is essential for Pup conjugation to the *ε*-NH_2 _ groups of lysines on several target proteins [16, 17]. The pupylated proteins are targeted for degradation by the mycobacterial proteasome [18]. Until recently, there were no indications that in archaea Ubl proteins perform functions other than cofactor biosynthesis, especially given that no archaeal E2-like proteins have been detected [7, 8]. Furthermore, there were some doubts that ThiS-like proteins in archaea are actually involved in thiamine biosynthesis because, unlike the bacterial case, the respective genes do not belong in the same gene neighborhoods with other thiamine biosynthesis genes, and an alternative pathway for thiamine biosynthesis has been proposed to function in archaea and eukaryotes [7, 19, 20]. 

In a striking recent development, the involvement of two Ubl proteins called SAMPs (small archaeal modifier proteins) in protein conjugation has been demonstrated in the halobacterium *Haloferax volcanii* [21]. Because SAMPylated proteins seem to accumulate in proteasome-deficient mutants and the targets of SAMPylation include ubiquitous metabolic and house-keeping systems of archaea, Humbard et al. hypothesized that the eukaryotic Ub system evolved from the SAMPylation machinery or a related archaeal system [21]. These groundbreaking results prompted us to perform an in-depth comparative genomic and sequence analysis of archaeal Ubl proteins and associated gene products; this analysis led to a number of functional predictions and a shift of the perspective on the likely ancestral functions of Ub-like proteins.

## 2. Materials and Methods

The recent update of the arCOG database [22] that includes 70 complete archaeal genomes (ftp://ftp.ncbi.nih.gov/pub/wolf/COGs/arCOG/) was used for the analysis of phyletic patterns of the relevant genes. The same database was also used for sequence retrieval. The NCBI Refseq database [23] was used for retrieval of information on genomic context. Protein sequence database searches were performed using PSI-BLAST [24] with an inclusion threshold *E*-value of 0.01 and no composition-based statistical correction. Additional sequence database searches were performed using the HHPred program which includes secondary structure prediction as part of the search [25]. The PSI-BLAST and HHPred searches allow prediction of protein fold through similarity to proteins of known structure. 

Multiple alignments of protein sequences were constructed using the Promals3D program [26], followed by a minimal manual correction on the basis of local alignments obtained using PSI-BLAST [24]. Protein secondary structure was predicted using the PSIPRED program that constructs multiple alignments of the query proteins with their homologs (whenever available) and employs these alignments for prediction [27]. Maximum likelihood (ML) phylogenetic trees were constructed by using MOLPHY program [28] with the JTT substitution matrix to perform local rearrangement of an original Fitch tree [29]. The MOLPHY program was also used to compute RELL bootstrap values from 10,000 replicates.

## 3. Results and Discussion

### 3.1. Ubl Proteins in Archaea and Their Classification

For the purpose of this paper, we define Ubl proteins broadly and in functional terms, rather than in terms of homology, that is, as small proteins that function as sulfur carriers in coenzyme biosynthesis and other metabolic reactions or that modify other proteins through conjugation that includes isopeptide bond formation. So defined, the Ubl proteins include the Ub homologs that adopt the *β*-grasp fold, the Pup-like proteins, and the additional proteins that are inferred to function via a similar mechanism on the basis of gene fusions, genomic neighborhoods and distinct sequence motifs (see below). 

In order to identify potential Ubl proteins in archaea as completely as possible, we employed two approaches. First, we performed PSI-BLAST searches against the archaeal subset of the NR database using as queries representatives of all previously identified Ubl protein families [1, 7, 8]. All proteins identified by these searches were linked to the updated arCOG database (see [22] and Section 2). The list of arCOGs that encompass potential Ubl proteins is given in Supplementary Table S1 available at doi:10.1155/2010/710303. This search allowed us to detect a few missing members of Ubl protein families, including a ThiS-like protein (NEQ520) in *Nanoarchaeum equitans*, an organism that was not previously noticed to encode Ubl proteins. The second approach was based on the identification of C-terminal motifs in multiple alignments of arCOGs. It has been shown that Ubl proteins (both *β*-grasp proteins and Pup-related proteins) possess a functionally essential double glycine (GG) motif at the C-terminus [1, 7, 8, 21]. Additionally, we noticed that one of the *β*-grasp related arCOGs from Halobacteria (arCOG00539) contains a double cysteine (CC) C-terminal motif. So we reconstructed consensus sequences for multiple alignments of all arCOGs and searched families that consisted of small proteins (<200 aa) with a conserved GG or CC C-terminal motif. Altogether we identified 8 arCOGs that met these criteria: 6 of which belong to the *β*-grasp fold, the 7th one (arCOG06308) possesses a TATA-binding protein- (TBP-) like fold (these proteins contain a C-terminal GG motif and are unique to Halobacteria), and the 8th one is an uncharacterized family (arCOG08988) with a “CC” C-terminal motif that is also specific to Halobacteria. The proteins in the latter family are predicted to possess a pattern of secondary structure elements (helix-helix-*β*-strand) that is clearly distinct from the *β*-grasp fold or the TBP-like fold but resembles the Pup domain [7, 8]. The phyletic patterns of all these arCOGs show that, among Archaea, Ubl proteins (primarily, the *β*-grasp domain proteins) are missing only from the genomes of several methanogens, namely, *Methanococcus jannaschii*, *Methanopyrus kandleri* and *Methanococcus aeolicus*.

**Figure 1:**
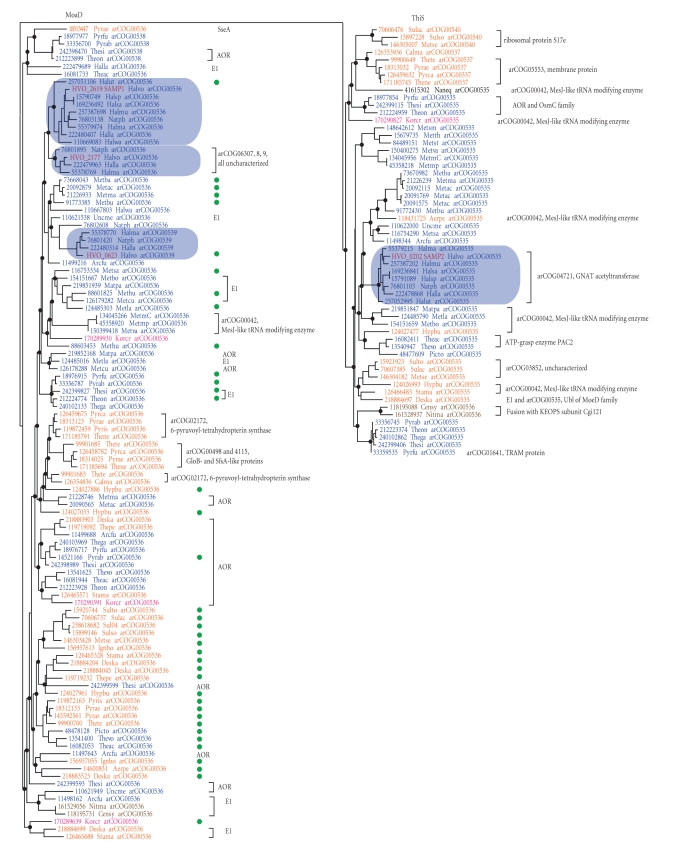
The phylogenetic tree and gene associations for the archaeal Ubl proteins of the *β*-grasp fold.

We analyzed all arCOGs that include *β*-grasp fold Ubl proteins by constructing a multiple alignment (Supplementary Figure S1) and a phylogenetic tree (Figure 1: The maximum likelihood tree was reconstructed using MOLPHY program [28] from 76 informative positions in the multiple alignment. The RELL bootstrap values are indicated for selected major branches: the branches supported at ≥50% are marked by black circles. The sequences are denoted by their GI numbers, abbreviated species name, and arCOG number to which this sequence has been assigned in arCOG database. Color codes for sequences are given as follows: blue—euryarchaea; orange—crenarchaea; brown—thaumarchaea; pink—korarchaea; black—*Nanoarchaeum equitans*. Major haloarchaeal branches are shaded. Proteins analyzed in the recent study of SAMPylation [21] are denoted by* Haloferax volcanii* protein identifiers and colored red. For the MoaD subtree, the expected associations with one or more MoCo biosynthesis genes are shown by green circles. Other gene neighbors are indicated on the right side of the tree (red) by indication of gene name, by full protein name, or by arCOG. Genes associated with Ubl are the following: E1-Ubl activating enzyme, ThiF/HesA family; AOR, tungsten cofactor containing enzyme aldehyde ferredoxin oxidoreductase; SseA, Rhodanese-related sulfurtransferase; GloB, glyoxalase; SfsA, sugar fermentation stimulation protein; OcmC, peroxiredoxin.). In this case, a highly reliable tree topology could not be obtained owing to the small size of the Ubl proteins resulting in a small number of informative positions. This caveat notwithstanding, the tree consisted of the two major previously established branches that correspond, respectively, to the ThiS and MoaD families [7]; moreover, the topology is reasonably compatible with the archaeal taxonomy and with the classification of the Ubl protein derived from the arCOGs (Figure 1). Therefore, this tree provides a useful framework for classification and potential functional inferences. The MoaD branch includes almost twice as many proteins as the ThiS branch. Several lineage-specific duplications are traceable in the MoaD branch including Crenarchaea- and Halobacteria-specific duplications. Several cases of likely horizontal gene transfer are also noticeable, for example, several euryarchaeal branches within the crenarchaeal part of the MoaD branch and, conversely, some crenarchaea embedded within the euryarchaeal part of the ThiS branch. The proteins in arCOG00540 that is specific to Sulfolobales, which so far have not been annotated as Ubl proteins, and those in arCOG00537 that is specific to Thermoproteales appear to cluster within the ThiS branch, pointing to additional duplications in crenarchaea. The tree also reveals a probable error in arCOG assignments for Thaumarchaea because two Thaumarchaeal proteins (GI: 161528937 and GI: 118195088) belong to arCOG00535 rather than arCOG00536. Given the diversity within both branches in the Ubl protein tree, it seems most likely that the last archaeal common ancestor (LACA) encoded at least two Ubl proteins with the *β*-grasp fold that represented the ThiS and MoaD families.

### 3.2. Gene Context and Domain Fusion Analysis for Ubl Proteins

Gene context and domain fusion analysis are central tools of inference under the “guilt by association” approach that is broadly used for prediction of functional connections for uncharacterized genes [30–33]. Most domain fusions can be automatically retrieved from arCOGs because the algorithm of arCOG construction includes splitting proteins into domains unless a fusion is conserved to the extent that it dominates the corresponding arCOG [22]. To analyze neighborhoods we retrieved three upstream and three downstream genes for each Ubl gene from a representative set of archaeal genomes (Supplementary Table S2) and identified the most common gene associations (Figure 1, Table 1, and Supplementary Table S3). Generally, we observed the same trends that have been pointed out previously [7, 20]. Most of the genes from the MoaD subfamily in archaea are associated with MoCo biosynthesis enzymes and the gene for aldehyde ferredoxin oxidoreductase (AOR) which utilizes the tungsten cofactor (a derivative of the molybdopterin cofactor). Like in bacteria, many MoaD-family domains are fused to the MoaE enzyme which is responsible for sulfur transfer to activated MoaD-like protein. We also confirmed the absence of contextual association of ThiS genes with any of the genes for thiamine cofactor biosynthesis. 

In addition, we identified several strong connections that have not been noticed previously, partly, because recently sequenced genomes help us to ascertain the evolutionary conservation of these associations. Mostly, these new associations are links between ThiS family genes and genes for proteins involved in translation. The most notable case is the association with PP-loop family ATPases that catalyze various tRNA modifications. In particular, the connection with the MesJ protein (arCOG0042) recurs in several archaeal lineages (Figure 1). The MesJ protein is nearly ubiquitous in prokaryotes and, in bacteria, is responsible for lysidine formation [35]. 

Recently, a tRNA modification pathway in yeast and in the nematode *Caenorhabditis elegans* that includes the Ubl protein URM1, two PP-loop ATPases (Nsc6p and Ncs2p), and two additional enzymes whose orthologs in bacteria are involved in thiamine biosynthesis (E1-like protein and rhodanese) has been characterized [36–38]. It has been shown that URM1 acts as a sulfur carrier protein for thiolation of uridine in the wobble position of some tRNAs; this modification results in an increased translational fidelity, in particular, preventing frame shift errors [37, 39]. Strikingly, three proteins that are homologous to URM1 pathway components (HVO_0558, arCOG01676; HVO_0025, arCOG02019; HVO_0580, arCOG00042) are SAMPylated with both SAMP1 and SAMP2 in *H. volcanii* [21]. The HVO_0580 protein, which is the ortholog of Nsc6p and a member of arCOG00042, is SAMPylated only with SAMP2 (HVO_0202), a ThiS family protein. Our observations complement these results and suggest that, even in those archaea where there is no genomic association between Ubl and PP-loop ATPases of arCOG00042 genes (which is the case in Halobacteria), these proteins function in concert. 

In Thermococcales, several Ubl genes are associated with genes encoding peroxiredoxins of the OcmC family (Figure 1), and indeed, a highly similar homolog of these proteins accumulates in proteasome mutants and is SAMPylated in *H. volcanii* [21, 40].

Several representatives of Sulfolobales encode a distinct family of Ubl proteins (arCOG00540) that are most similar to the eukaryotic URM1 family (Supplementary Figure S2) and therefore can be predicted to be involved in a URM1-like pathway. These *Sulfolobus* proteins are encoded in a distinct neighborhood which also includes genes for the ribosomal protein S17, an uncharacterized small protein of arCOG07188, a distinct membrane-associated HerA-like ATPase of the SSO0283 family [41], and a gene for an HSP60 family chaperonin, a thermosome subunit [42], which is transcribed in the opposite direction compared to the rest of the above genes (Table 1). Considering the data on SAMPylation of proteins encoded by genes adjacent to Ubl genes, it seems likely that the URM1 homologs in Sulfolobales regulate translation, proteolysis, and/or cell division through SAMPylation of, respectively, S17, HSP60, or HerA proteins, in addition to or instead of functioning in tRNA modification. 

Another notable observation is the fusion of a Ubl domain with the KEOPS complex subunit Cgi121. This fusion is conserved in all available genomes of Thaumarchaea (formerly known as mesophilic Crenarchaea [43]. The KEOPS (kinase, endopeptidase, and other peptides of small size) complex consists of 5 subunits (the names are those of the respective yeast genes that have been studied in most detail): Mn2+-dependent serine/threonine protein kinase Bud32p, ATPase of the ASKHA family (Kae1p), and three additional subunits: Pcc1p, Gon7p, and Cgi121p whose functions remain unclear. KEOPS complex has been shown to be involved in telomere maintenance and transcription in yeast [44–47]. The orthologs of the Kae1 and Bud32p subunits are present in all Archaea, the Pcc1p ortholog is missing only in a few archaeal genomes, and the Cgi121p ortholog is absent in Sulfolobales/Desulfurococcales and Nanoarchaeon. Taken together, comparative-genomic findings suggest that the counterpart of the KEOPS complex performs an essential function in archaea. The structure of this complex has been solved but the details of its functioning are still scarce although there are indications that it is critical for the maintenance of genome integrity in archaea [45–47]. The gene for the Pcc1 subunit shows a strong genomic association with genes that encode subunits of the archaeal exosome, the RNA degradation machine [48, 49]. Furthermore, the exosome genes themselves are associated with genes for proteasome subunits suggesting that RNA and protein degradation in archaea are tightly coordinated [48]. Very recently, it has been shown that in bacteria homologs of the KEOPS complex subunits are required for a distinct, widespread tRNA modification, the formation of N6-threonylcarbamoyladenosine (t6A) [50]. These findings suggest the possibility of regulation of the KEOPS complex by SAMPylation or coordinated functioning of the KEOPS complex, along with the Ubl-based system, proteasome, and exosome, in RNA and protein turnover control in archaea. Interestingly, the gene for the Cgi121-Ubl fusion protein is apparently cotranscribed with a gene for the ribosomal protein S17 in *Nitrosopumilus maritimus* and some other unfinished genomes of marine Thaumarchaeota, resembling the gene neighborhood in Sulfolobales described above.

The emerging trend of the association of Ubl proteins with genes involved in key information processing function in archaea suggests that several less frequent associations seen in a variety of different genomes also merit attention. For example, in two Thermoplasma genomes, the genes for ThiS family proteins are associated with the gene for the proteasome assembly chaperone PAC2 (Figure 1). In Pyrococci, ThiS family genes are associated with RNA-binding TRAM domain (Figure 1). Proteins containing TRAM domains are common in archaea; in particular, it is notable that a TRAM domain is fused to the essential enzyme 2-methylthioadenine synthetase that is involved in the thiolation of both tRNA and ribosomal proteins in bacteria [51–53]. In this case, again, the Ubl protein might possess a dual function: it could be involved in thiolation of tRNA (and/or ribosomal proteins) as a sulfur carrier or could regulate this process by SAMPylation or both. Finally, the only Ubl protein in Nanoarchaeon is located in the neighborhood of several informational genes including the proteasome alpha subunit and tRNA modification enzymes (Supplementary Table S2).

Surprisingly, it appears that either the functional specificity of Ubl proteins from different subfamilies can be easily switched or functional flexibility is an intrinsic feature of these proteins. For instance, the two functionally characterized SAMP proteins of *H. volcanii* belong to the two distinct branches of archaeal Ubl proteins, ThiS and MoaD (Figure 1). This hypothesis seems to be further supported by gene context and the dendrogram analysis, in particular, the association of Ubl proteins of the MoeB family with tRNA-modifying PP-loop ATPases and association of the ThiS family genes with the AOR enzyme (Figure 1).

### 3.3. Gene Context and Domain Fusions of E1-Like Enzymes

All known pathways involving Ubl proteins require E1 enzymes which activate these proteins via adenylation of the carboxy-terminal glycine residue of the Ub/Ubl polypeptide [54]. E1 enzymes possess a core Rossmann-fold ATP-binding domain [5]. Four distinct families of E1-like enzymes have been identified in archaea, namely, MoeB/ThiF/MOSC3 like, MJ0639-like, PaaA-like, and GodD-like enzymes [5] which in arCOGs are assigned to arCOG1676, arCOG1677, arCOG4786, and arCOG02882-2883,5002, respectively. However, PaaA and GodD-like enzymes are probably not involved in pathways that rely on Ubl proteins [5] and therefore are not considered here. Representatives of arCOG1676 are present in most archaea with the exception of the same methanogens that lack Ubl proteins (see above). However, all these methanogens encode a representative of the closely related arCOG1677 (Supplementary Table S1). The reconstructed phylogeny of arCOG1676 shows that the major euryarchaeal branch is well separated from the major crenarchaeal branch (Supplementary Figure S3). Some euryarchaea seem to have acquired from different bacterial sources additional E1-like enzymes; in Thermoplasma, these enzymes apparently have replaced the ancestral form. 

Most of the archaeal E1-like enzymes possess the same domain architecture (E1 core and a TBP-like C-terminal domain) as most of the bacterial homologs. There are also several other telling fusions shared with bacteria: Ubl-E1-TBP in Thaumarchaeota and Jab-E1 in methanogen RC1 (Jab is a predicted protease and/or DUB—see below). In addition, a unique architecture, with a small C-terminal small domain containing two conserved cysteines, is seen in *Sulfolobus* genomes. Analysis of gene neighborhoods for arCOG01676 did not reveal any new strong functional links. We detected many associations with Ubl-like genes and fewer links with enzymes of MoCo biosynthesis, thiamine biosynthesis enzyme ThiI, and cysteine synthase, all of which have been described before (see [5] and Supplementary Tables S2 and S3). However, it should be emphasized that the essential function of ThiI-like enzymes in prokaryotes is 4-thiouridine (S4U) modification of tRNAs [55], so it seems plausible that in archaea, which apparently synthesize thiamine via a distinct pathway [7, 19, 20], tRNA modification is the only function of ThiI. Furthermore, recently it has been shown that E1 enzymes and Ubl-proteins are also involved in thiolation of tRNA in Thermus thermophilus [56]. Thus, the same function can be proposed for at least some of the E1-MoaD associations seen in archaea. 

Interestingly, several representatives of the second E1-like family (arCOG01677) in methanogens are located in a conserved neighborhood which includes a gene for PP-loop superfamily enzyme, a predicted subunit of tRNA(5-methylaminomethyl-2-thiouridylate) methyltransferase (arCOG00037) [57]. However, the strongest potential functional association of arCOG1677 family genes remains enigmatic. In most methanogens, these genes are associated with genes for arCOG04865, which is homologous to the C-terminal domain of CinA, and arCOG04454, a NIF3 homolog (Table 1). In bacteria, CinA is a competence-induced gene often located in the same operon with RecA [58]. The NIF3 gene encodes a conserved metal-binding regulatory protein whose exact function remains unknown [59]. Given that arCOG01677 genes are never associated with genes for Ubl proteins, it seems unlikely that this group of E1-like enzymes is functionally linked to Ubl-dependent pathways.

### 3.4. Gene Context and Domain Fusions of Jab Proteases and Rhodanese-Like Enzymes

Metal-dependent proteases of the Jab family that in eukaryotes function as the primary proteasome-associated DUBs [4, 60, 61] and rhodanese-related enzymes that are involved in sulfur transfer reactions together with Ubl proteins [62] show similar but not identical distributions in archaea (Supplementary Table S1). These proteins are missing in many crenarchaea and methanogens. In archaea, the homologs of Jab proteases are rarely associated with Ubl genes or other genes involved in Ubl-related pathways. However, Jab genes are often associated with a gene for a cytidylyltransferase (Table 1 and Supplementary Tables S2 and S3), an association that could be of particular interest given that E2 and E3 enzymes required for Ub conjugation in eukaryotes have not been detected in archaea [6, 7]. A nucleotidyltransferase potentially could transfer an adenylated (activated) Ubl to a target protein, that is, perform the function of Ub ligase without sulfur-containing intermediates. The Jab protease is likely to function as a DUB similarly to its homologs in eukaryotes. Thus, it is tempting to propose the cytidylyltransferase-Jab tandem of enzymes as a candidate for an archaeal Ubl-conjugation/deubiqiutination system. 

Sulfur transferases of the rhodanese family catalyze the incorporation of sulfur into activated Ubl proteins via an intermediate persulfide. Rhodanese domains are often fused to ThiI like enzymes that also contain an N-terminal RNA-binding THUMP domain (Supplementary Tables S2 and S3). Many bacteria posses the same domain architecture and, as pointed out above, these enzymes are probably involved in tRNA modification. Only a few other associations of rhodanese-like proteins could be related to Ubl pathways (with AOR genes, for example), but most of other proteins of the rhodanese family are involved in either sulfur metabolism or redox pathways, which are likely Ubl independent.

## 4. Discussion

Comparative-genomic analysis indicates that most archaea encode members of two major groups of Ubl proteins with the *β*-grasp fold, the ThiS and MoaD families. The ThiS family genes are rarely found together with genes for thiamine and Mo/W cofactor metabolism enzymes but instead are often associated with various highly conserved and probably essential genes with functions related to translation, especially, tRNA modification. Thus, most if not all ThiS family proteins are predicted to function as sulfur carrier proteins for reactions similar to those recently characterized for the URM1 pathway in yeast [37]. In contrast, genomic associations suggest that the primary function of the MoaD family proteins is indeed the Mo/W cofactor biosynthesis. The absence of Ubl proteins and E1-like Ubl-activating enzymes of the arCOG1676 in such autotrophic archaea as *M. jannaschii* and *M. kandleri* and the absence of association of Ubl genes with thiamine biosynthesis genes (other than ThiI family enzymes which are probably involved in tRNA modification) is compatible with the existence of an alternative thiamine biosynthesis pathway in archaea.

Surprisingly, despite their apparent functional preferences, ThiS and MoaD family members appear to be interchangeable in pathways that employ Ubl proteins either as sulfur carriers or for protein modification. This possibility is born out both through analysis of gene associations for both subfamilies as described here and by the experimental data on the two SAMP proteins of *Haloferax volcanii* one of which belongs to the ThiS family and the other one to the MoaD family [21]. 

The most prominent associations revealed by comparative genomics for the archaeal Ubl proteins are with enzymes of tRNA modification. This finding leads to the hypothesis that the majority of the *β*-grasp Ubl proteins in archaea, at least those of the ThiS family, are involved in sulfur insertion steps of the biosynthesis of modified nucleotides. Given the ubiquity of a variety of tRNA modifications across cellular life [63], this is likely to be the ancestral function of the Ubl proteins that subsequently were recruited for other chemically similar reactions, such as MoCo and thiamine biosynthesis, as well as protein modification. This hypothesis is compatible with the role of the eukaryotic Urm1 protein in specific tRNA modification and with fusion of the Ubl domain to the KEOPS complex subunit Cgi121, given the requirement of KEOPS for the t6A modification. Experimental study of the involvement of Ubl proteins in tRNA modification appears to be an extremely promising research direction.

From a more general perspective, tRNA modification is undoubtedly a major mechanism of the quality control of translation [64, 65]. Considering also the association of another KEOPS subunit (Pcc1) with the exosome and the proteasome, it is tempting to view the Ubl proteins as general devices for protein quality control, both at the most fundamental level of translation fidelity and at the secondary levels of regulated protein and RNA degradation. In eukaryotes, the latter mechanisms assumed hugely diversified roles which required the evolution of the enormously complex Ub-centered signaling systems. 

The comparative-genomic analysis of the genes for Ubl proteins and the enzymes that appear functionally linked to them suggests that archaea might possess still uncharacterized Ubl-related functional systems. In particular, the association of the Jab protease with a cytidylyltransferase-like enzyme appears to be a candidate for a Ubl conjugation/deubiquitination system. In addition, archaea are likely to possess functional analogs of Ubl proteins that are structurally and hence evolutionarily unrelated to the *β*-grasp fold. This group includes small proteins of the TBP-like fold that bend at a GG doublet and are often fused to E1 family enzymes, in a strong indication of their Ubl-type activity, along with putative homologs of the bacterial Pup protein. 

In conclusion, the comparative-genomic analysis triggered by the seminal discovery of the SAMPylation reactions in *H. volcanii* reveals unexpected potential complexity of archaeal Ubl-centered systems and offers several directions for further experimentation, the most important of which arguably is the validation of the hypothesis on the involvement of Ubl proteins in tRNA modification. In addition, this analysis opens up an unexpected and potentially fundamental area of inquiry into the evolution of cells, namely, the ancestral connection between systems of protein quality control that operate at different levels.

## Supplementary Material

Supplementary Figure S1: multiple alignment of Ubl proteins. Supplementary Figure S2: multiple alignment of URM1-like proteins. Supplementary Figure S3: phylogenetic tree of the archaeal E1-like protein family (arCOG001676). The maximum likelihood tree was constructed using the MOLPHY program [28]; 245 informative positions were used for the tree construction. Color codes, sequence identifiers and species abbreviations are the same as in the Figure 1. Multidomain proteins are denoted by the names of domains, in the order from the Nterminus to the C-terminus, delimited by the “+” symbol (e.g., Jab + E1). The following genes and domains are associated with E1-like enzyme: MoaE, Molybdopterin converting factor, large subunit; MoaD – Ubl protein of MoaD subfamily; ThiI, Thiamine biosynthesis ATP pyrophosphatase; MoaB, Molybdopterin biosynthesis enzyme; TBP, TATA-binding protein (TBP)-like fold domain.

## Figures and Tables

**Table 1:** Components of Ubl-related pathways in archaea predicted in this paper^a^.

Reference arCOG	Representative of the reference arCOG	arCOGs identified by genomic context analysis	Lineage	Comment
Genomic associations of genes for UBL (*β*-grasp) proteins				

arCOG00540	70606476	arCOG01885 arCOG07188 arCOG00286	Sulfolobales	Predicted operon contains genes for 30 S ribosomal protein S17e, small uncharacterized protein, and a distinct membrane-associated HerA-like ATPase of the SSO0283 family. Thermosome beta subunit is a divergently encoded gene located within a conserved region which includes a variety of informational genes.
arCOG00537	18313032	arCOG05553	Thermoproteales	Membrane protein
arCOG00535	15791089	arCOG04721	Halobacteria	GNAT N-acetyltransferase
arCOG00535	15679735	arCOG00042	Many diverse archaea	tRNA(Ile)-lysidine synthase MesJ
arCOG00535	70607385	arCOG03852	Sulfolobales	Uncharacterized protein

Genomic associations of arCOG06308 (TBP-like fold protein with [GG] C-terminal motif)				

arCOG06308	76801892	arCOG06307	Halobacteria	Two small uncharacterized proteins and MoaD-like Ubl protein
		arCOG06309		
		arCOG00536		

Genomic associations of arCOG08988 (uncharacterized protein with [CC] C-terminal motif)				

arCOG08988	257373014	arCOG04404	Halobacteria	ComK-like protein, in bacteria, is involved in regulation of competence; in archaea, its role is unclear [34].

Genomic associations of genes for E1-like enzymes (ThiF/MoeB)				

arCOG01677	15679158	arCOG04863	Methanococcales	First two genes are distant CinA C-terminal domain homologs; next is an NAD-binding domain containing protein and an NIF3 homolog.
		arCOG04865		
		arCOG04864		
		arCOG04454		

Genomic associations of genes for the Jab protease				

arCOG01139	257387955	arCOG01222	Many diverse archaea	Cytidylyltransferase family protein

^a^The table lists genes (arCOGs) that are consistently found within genomic neighborhoods of genes that encode components of Ubl-related pathways. On the basis of theses associations and, in some cases, their domain content as well, the protein products of these neighboring genes are predicted to be functionally related to Ubl systems as well.

## Species Abbreviations

Aerpe: *Aeropyrum pernix* K1
Arcfu: *Archaeoglobus fulgidus*
Calma: *Caldivirga maquilingensis* IC-167
Korar: *Candidatus Korarchaeum cryptofilum* OPF8
Metbo: *Candidatus Methanoregula boonei* 6A8
Censy: *Cenarchaeum symbiosum*
Deska: *Desulfurococcus kamchatkensis* 1221n
Ferac: *Ferroplasma acidarmanus* fer1
Halma: *Haloarcula marismortui* ATCC 43049
Halsa: *Halobacterium salinarum* R1
Halsp: *Halobacterium sp.*
Halmu: *Halomicrobium mukohataei* DSM 12286
Halwa: *Haloquadratum walsbyi*
Halut: *Halorhabdus utahensis* DSM 12940
Halla: *Halorubrum lacusprofundi* ATCC 49239
Haltu: *Haloterrigena turkmenica* DSM 5511
Hypbu: *Hyperthermus butylicus* DSM 5456
Ignho: *Ignicoccus hospitalis* KIN4/I
Metse: *Metallosphaera sedula* DSM 5348
Metsm: *Methanobrevibacter smithii* ATCC 35061
Metin: *Methanocaldococcus infernus* ME
Metvu: *Methanocaldococcus vulcanius* M7
Metbu: *Methanococcoides burtonii* DSM 6242
Metmp: *Methanococcus maripaludis* S2
Metva: *Methanococcus vannielii* SB
Metla: *Methanocorpusculum labreanum* Z
Metcu: *Methanoculleus marisnigri* JR1
Metsa: *Methanosaeta thermophila* PT
Metac: *Methanosarcina acetivorans*
Metba: *Methanosarcina barkeri str. Fusaro*
Metma: *Methanosarcina mazei*
Metst: *Methanosphaera stadtmanae*
Matpa: *Methanosphaerula palustris* E1-9c
Methu: *Methanospirillum hungatei* JF-1
Metth: *Methanothermobacter thermautotrophicus*
Naneq: *Nanoarchaeum equitans*
Natph: *Natronomonas pharaonis*
Nitma: *Nitrosopumilus maritimus* SCM1
Picto: *Picrophilus torridus* DSM 9790
Pyrae: *Pyrobaculum aerophilum*
Pyrar: *Pyrobaculum arsenaticum* DSM 13514
Pyrca: *Pyrobaculum calidifontis* JCM 11548
Pyris: *Pyrobaculum islandicum* DSM 4184
Pyrab: *Pyrococcus abyssi*
Pyrfu: *Pyrococcus furiosus*
Pyrho: *Pyrococcus horikoshii*
Stama: *Staphylothermus marinus* F1
Sulac: *Sulfolobus acidocaldarius* DSM 639
Sulso: *Sulfolobus solfataricus* P2
Sulto: *Sulfolobus tokodaii* str. 7
Thega: *Thermococcus gammatolerans* EJ3
Theko: *Thermococcus kodakarensis* KOD1
Theon: *Thermococcus onnurineus *NA1
Thesi: *Thermococcus sibiricus* MM 739
Thepe: *Thermofilum pendens* Hrk 5
Theac: *Thermoplasma acidophilum*
Thevo: *Thermoplasma volcanium*
Thene: *Thermoproteus neutrophilus* V24Sta
Thete: *Thermoproteus tenax*
Uncme: Uncultured methanogenic archaeon.
