# Supplementary material for: Archaeal Ubiquitin-Like Proteins: Functional Versatility and Putative Ancestral Involvement in tRNA Modification Revealed by Comparative Genomic Analysis
Source: Archaea. 2010 Sep 26;2010:710303. doi: 10.1155/2010/710303 (PMC2948915; doi:10.1155/2010/710303)
Supplement: Supplementary file 1 — Supplementary Figure S1: multiple alignment of Ubl proteins. Supplementary Figure S2: multiple alignment of URM1-like proteins. Supplementary Figure S3: phylogenetic tree of the archaeal E1-like protein family (arCOG001676). The maximum likelihood tree was constructed using the MOLPHY program [28]; 245 informative positions were used for the tree construction. Color codes, sequence identifiers and species abbreviations are the same as in the Figure 1. Multidomain proteins are denoted by the names of domains, in the order from the Nterminus to the C-terminus, delimited by the “+” symbol (e.g., Jab + E1). The following genes and domains are associated with E1-like enzyme: MoaE, Molybdopterin converting factor, large subunit; MoaD – Ubl protein of MoaD subfamily; ThiI, Thiamine biosynthesis ATP pyrophosphatase; MoaB, Molybdopterin biosynthesis enzyme; TBP, TATA-binding protein (TBP)-like fold domain. [file 710303.f1.pdf]

```
76802608           LTVEFHGT---LRDAA-----DGSSV---------TRQFGDD-----------------------------------QTLGAVLEALADDYE-------SLGPLVFGSDG-------------------------RIRSNV--AVAVN-GDP-VTGEPRRDRQLSDG-DRLMLAPGL--------------------------AGG  97
3dwm_chainA_p002   VTVSIPTI---LRPHT-----GGQKS---------VSASGD-------------------------------------TLGAVISDLEANYS-------GISERLMDPSSP-----------------------GKLHRFV--NIYVN-DED-VRFSGGLATAIADG-DSVTILPAV-----------------------------  89
76801103           VTCDIVGE---------------DTR---------ELDVDEA-----------------------------------ATYGDLLEAVGL-----------------------------------------------SSHEA--SVLVD-GSP-----VPEDRTV-DS-DSVRILRLI--------------------------KGG  66
257052995          VTVELVDE---------------ITR---------EFDIEPG-----------------------------------TTYGELLADFEV-----------------------------------------------SPHEV--SVLVD-DRP-----VPEDEAV-DA-EHVRIVRLI--------------------------KGG 102
257387202          VTVEIVGG---------------ETR---------EVEIAAG-----------------------------------DSYADLLAPTDL-----------------------------------------------SPHTV--SILVD-GTP-----VPEDQPV-ET-DHVRVVRLV--------------------------KGG  66
55379215           VTVEIAGE---------------DTH---------ELEVDAD-----------------------------------ATYGDLLAPVDL-----------------------------------------------SPHEV--SVLVD-GEH-----VPTDQPV-EH-DHVRVVRLI--------------------------KGG  66
15791089           VTLSVVGG---------------DTH---------EFTVTTAADTE---------------------------PGPPTTYADLLADVGL-----------------------------------------------SPHEV--AVMVD-GSP-----VPEDHPV-DA-DFVRVLRLI--------------------------QGG 174
169236841          VTLSVVGG---------------DTH---------EFTVTTAADTE---------------------------PGPPTTYADLLADVGL-----------------------------------------------SPHEV--AVMVD-GSP-----VPEDHPV-DA-DFVRVLRLI--------------------------QGG  74
222478868          VTVEVVGE---------------GTA---------EYDLPDD-----------------------------------ATYADLIREAGY-----------------------------------------------HPQEA--SALVD-GSP-----VPGDRLV-DA-ESVRLLRLI--------------------------KGG  72
76801892           -----------------------------------TRTFRG------------------------------------ISKRLAISYLEGLGG-------EHNGESR-----------------------------VEGDGW--AATLD-ART-----VSPAGSIRL--SEVEIRFEGDRDELDPVI--------EAFERKAVRAGG  84
55378772           -----------------------------------ERSFRG------------------------------------ISVRAAIGYLENLGG-------EQRGEAT-----------------------------VEGDGW--AATLS-EEK-----VAIGPSLQL--NEVTIQFDGDPETLEPLI--------EKFAQKAMRAGG  84
110667803          -EITVYGP---LRTAA-----DGKVL---------TVATGSSTDGTDKNAANSNTIS----EGSESIDTSSVSASSPETVSELITMIAETYP-------QTKSHLFDCEG-------------------------TLRPSV--RILVN-GNK-----ASPETQLDQS-ADIQLFPAM--------------------------RGG 127
222479962          EEVAYRGI---SRRLA-----AHYLVNLGGELIGTDDPAEATRVDGDGWRVGLSAETVKAAGSISLTEVTAEFAGEPEALAELLP--------------KYRQKAMR------------------------------------------------------------------------------------------------AGG 116
14600831           VELRLYTI---LKDAA-----GAETV---------RVPCPPG----------------------------------GVTLGEALREAARTSG-------GLERALDA-----------------------------VSWEV--YGLLDDGSR-----LALEDRVACG-SRVHVIPPP-------------------------SGGG  91
222479689          ATIKLPAV---LVGGS-----STSEV---------DVDG--------------------------------------ETVRELFENHADEHG------SELRDSVIKDG--------------------------EIKEFI--NVYVE-GTP----VDSLDAEVPDD-AQVRVIPAA--------------------------SGG  87
55378770           VEVRCTGH---VRRVV-----GEPAL---------SYTFEG------------------------------------NTLRDFLDAFFQEYD-------VGDMLIAETEADATTEGWAPEIADLPGDWAKNPEGEQTRCYA--RVAVN-GEF-NEHLDGLDTELETG-DRIGLLYPF-------------------------IFCC 133
222480314          VTVRCTGH---VRTEL-----GKYEF---------EYTFEG------------------------------------DTLREFLDELFVEYP------ELQEMLIAESEEESTHSGWAPTPEELPGTWTKNPVGEQTIAYA--RILVN-GHF-NENEKGFDTELEEG-DRVALVYPF-------------------------MFCC 165
76801420           VTVRCTGH---VRDTV-----GTHEL---------EYTFEG------------------------------------RTLRAFLQAFFGDYD-------VEDLLIAETEAEATTSGWAPSDGDPPGAWAKNPEGEQTRSFA--RVAIN-GQF-NEHLDGLNTELADG-DRVSLLYPF-------------------------MFCC 145
161528937          ITVKLVGG---AKKSF-----STEKL---------EFEKSN------------------------------------ITIQELIDLLLETKP-------NDSPEL-------------------------------DTENI--LIAVN-GVD-SSALEGKSTMIKND-DVVSIIPVI--------------------------HGG  85
118195088          ITVRLLGG---ARKAF-----GAETV---------EVEGDK------------------------------------MTVRELLDSLKSRNP----------RDL-------------------------------DLDSL--LVAIN-GAD-SAVS-----------------------------------------------GG  82
73670982           VHITIQAG-------------EISEQ---------TVEVAES-----------------------------------ATYEDLLNTLDI-----------------------------------------------NQETV--LVLNG-GNA-----VPLDGTVSS--DRLTILRVV--------------------------TGG  70
110622000          LTVILHAG-------------RKTET---------QVDLPEG-----------------------------------SSYFDLLRELKV-----------------------------------------------NPETV--VVFKN-GIP-----VAFDSVV-EG-GTVEVMRVV--------------------------SGG  71
116754290          VSVRIFAG-------------VESER---------QLVLPEG-----------------------------------TSYYEILDLLGI-----------------------------------------------NPETV--AITRD-GVP-----VPFNDLVLP--GEIEIIRVV--------------------------SAG  69
21226239           VHITIQAG-------------EIVEQ---------TVEVAET-----------------------------------ATYEDLLDELNI-----------------------------------------------NQETV--LVLNG-GNA-----VPLDGAIGS--DKLTILKVV--------------------------TGG  70
20092115           LYITIQAG-------------EISEQ---------TVEVAES-----------------------------------ATYEDLLDTLNI-----------------------------------------------NQETV--LVLNG-GNA-----VPLDGAINS--DRLTILKVV--------------------------TGG  70
20091769           VHVRIQAG---ALCGAM---EEISEQ---------TVEIGEN-----------------------------------LTYENLLDILGI-----------------------------------------------NDGTV--IVLNE-GNA-----VPLDGIISS--DKLTVMRVV--------------------------SGG  77
20091575           VHVQIQAG---ALCGAM---EEISEQ---------TVEIGEN-----------------------------------FTYENLLDILGI-----------------------------------------------NEGTV--IVLNE-GNA-----VPLDGIISS--DKLTIMRVV--------------------------SGG  77
91772430           IKVKIFPD-------------TPDGQ---------AINVPDG-----------------------------------STYEDLLNVMDI-----------------------------------------------NQELV--ILLND-GKA-----VPIDDVVIE--GTVTILKAI--------------------------SGG  70
11498344           LKIKFVGF-------------DQKEV---------EVEVNG------------------------------------QRYSEILESLGI-----------------------------------------------NPETV--VVVKD-NIP-----VPVDDVA-EG-GEVKVVRVI--------------------------SGG  67
15679735           IGMKFTVI------TD------DGKK---------ILESGAP-----------------------------------RRIKDVLGELEI-----------------------------------------------PIETV--VVKKN-GQI-----VIDEEEIFDG-DIIEVIRVI--------------------------YGG  70
84489151           --MTIKLI------NK------KDIK---------DIEISEN-----------------------------------TTIADILKKEEI-----------------------------------------------PIETV--VVKLN-GDT-----VTEDEKVKNG-DELEIIKVI--------------------------YGG  66
154151659          -HRALLPS---FRESMRVLLPDQGIR---------VIDWGP------------------------------------GTVAALLQELGI-----------------------------------------------NPLEV--MVARN-GAL-----VPEQAAIGGD-DEIRIIRIA--------------------------HGG  76
119872163          IVIKYFSV---LRDIT------GKIRE---------ELDLKNG-----------------------------------TSMSQLIEWFFKTYP-------KAEVF---------------------------------REEL--LILVN-GRT-----VDNNYILKDG-DEVAFMPPVS-------------------------GGG  82
99900700           LKLKYFSA---LRDIT------GKVSE---------ELEVSGD-----------------------------------LTLGDLVKWFFEKYP-------KALAY---------------------------------KDDV--IFLVN-GRN-----ATESYLLRDG-DEVAVMPPVS-------------------------GGG 108
171186442          IVLKYFSA---LRDIT------GKARE---------ELSMEDG-----------------------------------ARLAQVIDWFFNMYP-------RAEVF---------------------------------REEL--LVLVN-GRA-----VDGTYVLQDG-DEVAFMPPVS-------------------------GGG  83
126460739          VLLKYFSA---LRDIT------GSARE---------EVAFEGP-----------------------------------VTVSDLLTWFFKKYP-------KAEAF---------------------------------REEL--VVLVN-GRA-----VEGSYVLKDG-DEVALMPPVS-------------------------GGG 102
18312133           IHIKYFSA---LRDIT------GKTSE---------TIEIPNG-----------------------------------YTLGDVINWFFKNYP-------KAEVF---------------------------------KEEL--LVLVN-GRS-----LDWSYELKEG-DEVALMPPVS-------------------------GGG  83
145592561          IRVKYFSA---LRDIT------GKTSE---------DLEMPDG-----------------------------------STLGDLINWFFKQYP-------KAQAF---------------------------------KEEL--LVLVN-GKS-----LDWSYVLNGG-DEVAIMPPVS-------------------------GGG  83
15920744           IRLRYFSF---IQDFT-----NKSSE---------EIETECK------------------------------------TVECLLEQLSSLYGDEFLRIIKNGLG---------------------------------SVRV--TILVN-GKA-----NVNN--INDG-DEIAILPPP--------------------------SGG  85
70606737           IKVRYFAL---LRDYT-----KKNEE---------IIETECK------------------------------------DVACLVSQLEKVYGKEFGRVVREGFG---------------------------------PVRI--VILVN-GKV------DNE--IREG-DEVALLPPP--------------------------AGG  84
146303428          VKVRYFAL---VKDIT-----GKDSE---------EIDTRCS------------------------------------KLNCLLDELVEKYGPRFKALLQGQVS---------------------------------GVKV--FFLVN-GKM-----NVEE--IREG-DEVAILPPP--------------------------AGG  85
15899146           VKVLYFAF---IKDIT-----HKSNE---------VLETECE------------------------------------TVDCFIEQLGKIYGSELVNFLKSGIN---------------------------------GIKV--SILVN-GSA-----STKN--IKDG-DEVALLPPP--------------------------SGG  86
238618682          VKVLYFAF---IKDIT-----HKSNE---------VLETECE------------------------------------DVGCLIEQLGKMYGNELVNFLKNGIN---------------------------------GIKV--SILVN-GSA-----STKN--IKDG-DEVALLPPP--------------------------SGG  86
21226933           VKVKLFAN---LREAA-----GTPEL---------LLSG--------------------------------------EKVIDVLLSLTEKHP-------ALKELIFEKNDGED-------------------ESPVLCASI--NVLIN-GNN-VRHLEGLDTDLKDS-DEIGVLPPV--------------------------SGG  97
18313447           IYVKFLGP---YMEKAG----TSDDE--------VEIDN--------------------------------------EITLGELLELMARKY------NITELLNFPNSQ-----------------------------EV--AVLVN-GTQ-ADD---VKKRLSDG-DEVVILPAL--------------------------AGG  88
20092879           VKVKLFAN---LREAA-----GTPEL---------PLSG--------------------------------------EKVIDVLLSLTDKYP-------ALKYVIFEKGDEKS-------------------EILILCGSI--NILIN-GNN-IRHLEGLETLLKDS-DEIGILPPV--------------------------SGG  97
73668043           VKVKLFAN---LREKA-----GTSEL---------QLSG--------------------------------------EKVIDVLLALTGKYP-------ELKNLIFEEPEGES-------------------EAPVMCGSI--NVLIN-GNN-VRHFDGLDTLLSDA-DELAVLPPV--------------------------SGG  97
257051106          VSWKLFAT---LREAA-----GQAEV---------QVAVGTD-----------------------------------GTVADALEELLEEYP-------TLETDALDGNE--------------------------LRAHI--SVVHN-GVR-IDEPLASDRELADG-DELALLPPL--------------------------SGG  92
11498162           IKVRLPAS---INQ---------GDL---------EIDAEK------------------------------------LTVNQLIALLSQITG-------KDLEKILKRDG-------------------------ELSPFI--SIFVN-GRN-VRYAEGLETEVRSG-DEVSIVPTV--------------------------AGG  88
110621949          DRKSLEFQ---FRPG--------------------------------------------------------------MTMRDLFAELSQFGR-------QGFEKAIYDPRT-----------------------GRMNEYL--AVFVN-SRE-IRSLDGLDTRLTGG-DTVTIMPPM--------------------------AGG  86
126465328          IKVKYMLW---LREKI-----GIEQE---------EFKLDRE-----------------------------------TTLENLVRMIILKHR-------EAEKYLSNIF--------------------------SDNNPF--IILVN-GRP-----GRKNYILKDN-DLVTILPPV--------------------------SGG  88
119719232          LQVRYVSL---MKDKT-----GKEQE---------EIELDAE----------------------------------EPTLEDLLRIL-------------EEKYGVPML--------------------------SGEEEM--LVLVN-GRR-----SGLKEKLSDG-SQVVIAPPV--------------------------SGG  83
161529056          ITFTIPSV---LNHGG-----GEKKI---------EIPA--------------------------------------DSLQDVFTKISEQMG------DDFKRRVLEGDG-------------------------TPRSLI--NIYIN-GKN-AKFSSGMETALKDG-DEIYILPAV--------------------------AGG  92
118195731          ITFTVPPV----LNGG-----TEKKI---------PMSA--------------------------------------GTLKEAFDAVCKDMG------EDFGRRVLEKDG-------------------------TPRSLI--NVYVN-GKN-ARFSGWMDTAFKDG-DDVYLLPAV--------------------------AGG  91
218884204          VRVRYMLW---LESKA-----GTREE---------VIELNKG-----------------------------------STLRGLLEIVSERHP-------GLAKYISELL--------------------------QGTSQI--IVLHN-SKT---SGKGLDTVLMDM-DTVVLMPPV--------------------------SGG  90
218883523          ITIRSYSI---FSDFL------GNEV---------RIELDES-----------------------------------ITVGELLDYLKNTYS-------LP-----------------------------------SKPEP--IVLIN-GEI-----ASLDQQLRDG-DTVYIVPPF--------------------------SGG  78
146305007          VKVVLRGP---LVSIF-----SGNEF---------SVKG--------------------------------------DNLISILSKIDK-R-----------GIIVSDG--------------------------RIKPGY--LILVN-GMD-FRLLK--KEVLEDS-DTVDIIPIN--------------------------HGG  81
15897228           PKVILKGP---LISQF-----NFREI---------YVND--------------------------------------RELLRVLVKIDSKK-----------HLILNESN-------------------------QLKSGI--LILIN-GKD-WRLYR--NQLLNDN-DIIEIIPIN--------------------------HGG  83
70606476           VKIRLKGP---LATRL-----GRDEF---------VISLKA------------------------------------DNLLDILKELDKEE-----------KLLINGN--------------------------KIRSGY--ILLIN-GID-YRLLN---GKLKDG-DVVDILPIN--------------------------HGG  84
18976717           MQVKLFAT---LIEFT-----GKRKL---------EIHGP-------------------------------------KTVRELLDKLEEMFP-------GFKKEL--------------------------------EQGY--MILVN-GKN-IEHLQGLDTPLSDD-DTVSIFPPA--------------------------GGG  82
242398989          VRVRFFAT---LRNIV-----RKNEL----------ELQA-------------------------------------NTVGELLEKLYSKYP-------KIEKEL--------------------------------EKGV--VILVN-GKN-IEHLEKLNTKLKDE-DLVSIFPPV--------------------------GGG  82
240103969          VKVKVFAT---LIDIV-----GKRML---------EIGGV-------------------------------------KTVGELLDELDRRFP-------GVKKEL--------------------------------EDGY--LILVN-GKN-IEHLQGLETPISDD-DTVSVFPPA--------------------------GGG  83
14521166           VKVKVFAT---LIDII-----GRRIF----------EEEA-------------------------------------ENVEDLLNKIYSKYP-------KVREEL--------------------------------EEGY--IILVN-GHN-IEHLQGLRTKLKDG-DVISIFPPA--------------------------GGG  84
212223928          MKVKFYAT---FRELI-----GRKEI---------EVHDI-------------------------------------KTVGELIDYLAKHYS------PEIKREL--------------------LESPRVKEAGKAIDG---MILVN-GHN-VLHLKGLDTELKDD-DEVHIFPPA--------------------------GGG  94
126465571          VKIKVYMT---LPETLG---WKEKII---------ELPRDK------------------------------------STFRDLLDKL-EDIK-------KIKDDL-------------------------------EKKGWRLIILVN-GKH-IEFLNGMDTVLNDG-DEIAIFPPA--------------------------AGG  93
170290391          VEVRVYLT---LREKL-----GWKSK---------TLSLGRN----------------------------------KVKFSELLDELKDLKG------VLEDFG---------------------------------YENF--MILLN-GRN-IRLLDWLDTEVEEG-DSIDIFPPA--------------------------GGG  97
156937613          VKVQFFAF---LRDEI-----GKKED---------EFECDC------------------------------------SSPEELLEELFKRYP-------KLKEEDEQYK--------------------------RFGLEL--KVLVN-GRE-WKY---LDSLKGDE-VEVALFPPA--------------------------AGG  88
126178288          VKVRAFAM---LREII------PAEY---------DVKLPGG-----------------------------------STVGDLLDHLSTRHP-------GLGDALFTSAG-------------------------ELAPYV--NILTN-GRN-IHFSGGLETPLEDS-DTVALFPPA--------------------------GGG  92
18313123           IRVKFLAT---LYDLT-----GVMKT---------ELEVPQG-----------------------------------ITVKSLIDILNEKFP-------KLKSELLEEGN-------------------------QLKPMY--NILVN-GRA-VEWLKGLDTELKEG-DEVVFIPPA--------------------------AGG  93
99901685           VKVRIMAP---LYFGLDS--VDQIRL---------ELELPEG-----------------------------------STVEDVIDKIDSLYK-------GFKNKII-KNN-------------------------KIIDMH--DILVN-GRS-IDFLEGLKTKIKDN-DLIIIVSPF--------------------------GG-  94
124027033          ITVRLYAG---FHEAV-----GSTEL---------ELEVPKG-----------------------------------VTVLDVARILEEKFP-------GLRGRLVKDG--------------------------ELQDDV--LVLVN-GRS-IEWLEGVKTKLRSG-DRVAFFPPA--------------------------AGG  92
99901683           IRVRYLAT---LFDLM-----GTMRD---------VVEVPEG-----------------------------------ATVRDLIRAIDRKHN------GRLSKEIL-DGD-------------------------KLKDEY--NILVN-GRA-IDYLAGLDTRLKDN-DEVVFMPPV--------------------------GGG 100
171185791          VRVKFLAT---LYERT-----GVLKT---------EVEIPEG-----------------------------------SSVRDLIKILDSRFR-------GLESELLDESG-------------------------GLKPMY--NVLVN-GRA-VEWLNGLATALREG-DEVVFIPPA--------------------------AGG 100
119872459          IRVKFLAS---LYELT-----GVLKT---------ELDVPDG-----------------------------------ISIKELISLLSMRYR-------NLETEILDETG-------------------------NLKPMY--NILVN-GRA-IEWLNGLATKLKDG-DEVVFIPPA--------------------------AGG  93
126459675          LRVKFLAT---LYDLT-----GVLKT---------EVEVPDG-----------------------------------ITVRELIDVLDQKFG-------KLKAEIL-EGD-------------------------KLKPMY--NILVN-GRA-VEWLNGLETRLKDG-DEVVFIPPA--------------------------AGG  92
126354836          VTVKFLAL---FYEMA-----KTLRM---------ELNVPDG-----------------------------------ISVRDLLKIIDEKVN------PNISKTLLSDDS-------------------------GLREGY--NILVN-GRA-IDYVKGLDTVLKDG-DEVVLLPPI--------------------------DGG  94
126458782          VTVKIFGP---KYFGLPQ--YDVVTH---------VFDLPDG-----------------------------------ATVGDVVDLLEKMYP-------GLRGRILRGE--------------------------EIIPMH--DIWVN-GRS-IQFLQGLATRLKDG-DVVQLVPPF---------------------------GG  94
171185694          VVVKIYGP---KYFGIDR--YDVVTE---------VLEVDGE-----------------------------------PTVMDVLDKLEGRYP-------GLKRRLLSGD--------------------------VVAPMH--DVLIN-GRS-IAFLRGLTTALKDG-DVVQIVPPF---------------------------GG  94
18314025           VLVKIFGP---KYFGLDT--YDVITL---------VLDLKEG-----------------------------------ATVGDVMEELEKRYP-------GLRQKLLRGE--------------------------EIIPMH--DIWVN-GRS-IYFLQGLKTALAEG-DVVQIIPPF---------------------------GG  94
124027886          VKAVFIQE---FYELM-----GRFEL---------EIELAEG-----------------------------------ATVRDLIEYIDSQVK------PGFKSWILDEKG-------------------------EPRYPV--EIAVN-GRR-IDFLDGLETRLKDG-DRVLFSPRA-------------------------LFVV  95
218884699          ITVITLGR---ASDYT-----GRSMV---------ELELPEG-----------------------------------AMLRDAIRVLSEKTN-------PLLYERYIDG---------------------------HYIFV--TLVND-KPV-----LSPDTPLKDG-DRITLVTPE--------------------------MGG 106
242399593          VFVKLIAQ---VAEGI-----ERLEF---------WVFLEEN-----------------------------------STLGDLLKKLELE------------RKIKIE---------------------------WDKRNI--VILIN-GRS-AEFLGGVKAKLKDM-DKIVIMPVA--------------------------AGG  90
170290827          IKVSIGGE---------------------------ERTLPAN-----------------------------------TQLSDLYKILGF-----------------------------------------------SKWEI--LLLIN-GKP-----RSDDSYLEDG-DLVTWLRITLHE-------------------SPVSESR  73
170289639          MRIRFLGK---PKEIM------GGVI---------ELNLGR------------------------------------ATLREVLERLPED-----------VRKLITD---------------------------GEKLKM--IVLIN-GVS-AESKGGLDAPVSQD-DELTIMPEI--------------------------SGG  95
171185745          ARVKLAGV---LVALA------GGRA---------ELELEG------------------------------------STVREILGGLASVSQ-------KLYERVVGPDG-------------------------RPRADI--YIAVN-DVD-IRLLSGLSTAVKKD-DVVLILAYI--------------------------HPG  90
99900649           VKVILDGV---LVYLA-------GKR---------ELQVDA------------------------------------GSVRELIERLKDRP--------KLYERITRGPR------------------------GELAPDI--YIAVN-DVD-IRLLSGLDTPLGPE-DVVLIIAYI--------------------------HGG  89
18313032           PRVKLAGV---LVALA------GGRD---------EVVLEG------------------------------------ATVKEILSALSSISP-------KLYKRVVGEDG-------------------------RPRADI--YIAVN-DVD-IRLLSGLNTPVKKD-DVVLLLAYI--------------------------HPG  90
126459632          PRVKLAGV---LVALA------GGRS---------EFEVEG------------------------------------ATVREVLENLASVSK-------RLYERVVEPGG-------------------------RLRPDI--YIAVN-DVD-IRLLSGLATRVEKD-DVVLILAYI--------------------------HPG  90
126353936          VKVKFAGV---LVTFA-----GSETI---------DVGVKCG-----------------------------------STLRDLLRQLASVNP-------RLKRRLIEDD--------------------------GLWPGV--YVAIN-DTD-IRLLNGLDTRLNNG-DVVLFLSYI--------------------------HGG  96
21228746           INVKFLAS---IREIA-----GAHEM---------QFELNSG-----------------------------------YTVKDLLEFLELRFG-------AEFKEAAGKPF------------------------EDENPRL--RFLVN-GRD-IDYLRGPGTELKEG-DLVVLIPPV--------------------------GGG  94
20090565           IHVKFLAT---IREIT-----GKPEI---------ELEILPG-----------------------------------DTVGTALQALQARYG-------PEFKEATTGTT------------------------AGGIPKV--RFLVN-GRN-TDFLDGFETELKAG-DVMVFVPPV--------------------------AGG  94
148642612          FTLIFKDI-----------------S--------EERDIPKE----------------------------------SYTIKDLLNDLELS-----------------------------------------------SQTI--VPKQN-GEL-----VIEESEIHDD-DEIRLIQII--------------------------YGG  67
156937055          VKLKLYAM---YKDAL------GEAA---------ELELPEG-----------------------------------SKVADLKRKVLELLP-------------------------------------------EGYPEP--VVLKD-GRF-----AEDEEPLTEG--EYELVPPA--------------------------AGG  80
124027477          VRLNILG---------------NGVR---------EVEVAEG-----------------------------------ARVADVLREA-GLLS-------------------------------------------TEY-----VVVKG-GRV-----VAEDEPVADG-DELILYPVV--------------------------SGG  68
124485303          ITIRSFAK---FRELF------GEVN---------TVSVPEG-----------------------------------TSILGALLAFAKTQK-------DGMDELF-AGG-------------------------KLGSHI--ILMYN-RER-IDSDDAKTIHMAEG-DELVIYPPV--------------------------SGG  92
88603453           IRYQTYAR---FVEFF------GKEG---------DIEVIPG-----------------------------------TTVQGFLELLTGEDE-------KKQSLLFDENG-------------------------TVRRYV--IILKN-KDR-ILNEETDSTDLQDG-DELIIYPPV--------------------------SGG  92
88601825           VTIRCYAR---FRDAF------GEEQ---------TLDLPEG-----------------------------------STIHDAVRALAGDGP--------DAELLVDTAH-------------------------NIRSYV--MIMHH-DTR-ISPMEAESVPLADG-DLIILFPPV--------------------------SGG  91
126179282          VRVRTFAR---LREAI------GGDL---------TLEVPEG-----------------------------------ATMSRLLAVVAETSE-------QAGEALFEESG-------------------------ELRGYV--ILMHN-GRR-VRRAEAMTLALADG-DEVALFPPV--------------------------AGG  92
126466483          VIIR-------LVDG-------SKEW---------RIEVPEG-----------------------------------SSVRDVLASIGLISN-----------------------------------------------EY--VVVRN-GVV-ISEDE----EVRDG-DILILYPVV--------------------------SGG  68
11497643           VKVTLSAV--QFRSLV-----DYESRS-------FHLDLGEG-----------------------------------STLGDAVRAVEEMLS-------AEIEPLLE------------------------------RGNV--RIMMN-NSL-VDYQKEKNRVLNDG-DALIFITPI--------------------------SGG  91
170289930          VNVKFHAF---YKDVA-----GTDHV---------TFEMPEG-----------------------------------STVLDLLRAIEERFE-------GFRGKLIDRK--------------------------VGSRSY--ILVRR-GEW-----PGMEDALEDG-DEFSLFPPL--------------------------GGG  88
55378769           IELRFFAN---FREAV-----GQKTV---------HREYETD-----------------------------------LQAGDVLRQLSEEFT-------EMD---LFEDG-------------------------ELREYL--TILRN-GRD-IAHLDGLETPLEDG-DELSVFPPV--------------------------AGG  90
110621538          IKVKLFAN---FREAT-----KQKEV---------EISPEG------------------------------------DSVKAVIKGLVTKYP-------NIEPL-LFQDG-------------------------ALKPYV--NVLHN-GQT-VKGEEGLTAKIKDG-DEIALFPPV--------------------------SGG  91
```

```
91773385            VKIKLFAN---LREIA-----GVSEL---------ELEG--------------------------------------ENIQEILDILQNDHP-------QIQELIYDDDRG----------------------KKEIRAYI--NILIN-GNN-IQHLEGSDTVLNEG-DEIAIFPPV--------------------------SGG  112
11499216            VRVKLFAN---FREAA-----GVKEV---------EVEA--------------------------------------GTVGEVLQELVRRFP-------KLESL-FYEEG-------------------------RLRDYV--NIMVN-GRN---VRGDLNYPLSHT-DEVAIFPPV--------------------------SGG   86
48478128            IRIIYFAA---ARDRS-----GTDNE---------YIDYKG-------------------------------------SLTELKKIIYERHN-------DLKSMDLLFA--------------------------VNRRYV------------------DDCIIKDG-DEIAVFPPV--------------------------SGG   75
76803138            MEWRLFAN---LAEAA-----GTKRV---------EVDAAPG-----------------------------------DTFGDAFEQLLAAHP-------DLEAEVLDEDG-------------------------ELRDHI--RVLRNDRNP-FVSDDGFETTLEEG-DELALFPPV--------------------------SGG   92
257387698           MHWRLFAT---LAETA-----GEDRV---------SVDDA-------------------------------------ETVGEALDALLSAHP-------ALESEVLDDDG-------------------------SMVDHI--RLLHD-GDDPFAAGDGLDTPVSPG-DELALFPPV--------------------------SGG   90
55379974            MEWKLFAH---LRDAA-----DGQSV---------SVDIEGD-----------------------------------ATVETALDALLATRP-------ALAEEVLDENE-------------------------ELADHI--RVLVD-GEDPFAAGEGLATAVDEE-TELALFPPV--------------------------SGG   92
222480407           MEWKLFAD---LAEIA-----GGREI---------DVAAEPG-----------------------------------DTVGDALDALLEAHP-------DLRDRVL-DDG-------------------------EVANHI--NVLRN-GQN-VRQNGGMDTTLEAG-DELALFPPV--------------------------SGG   94
110669083           ITWRLFAD---VAEIA-----GTREK---------SIDIHTN-----------------------------------ATIEDAIDELLNDRP-------ALANRVLLDPGTK--------------------TETELATEI--NLLQN-GSD-----ANRDDQLTDG-DELALFPPV--------------------------TGG  118
15790749            MEWQLFAD---LSEVA-----GDDTV---------TVAVPTD----------------------------------DPTVQDALDALVADYP-------ALGDRVLAADG-------------------------SLAAHV--NVLVN-GVT--VDADDLDTAIAAG-DELAMFPPV--------------------------SGG  100
169236492           MEWQLFAD---LSEVA-----GDDTV---------TVAVPTD----------------------------------DPTVQDALDALVADYP-------ALGDRVLAADG-------------------------SLAAHV--NVLVN-GVT--VDADDLDTAIAAG-DELAMFPPV--------------------------SGG   91
76801893            LALRFFAT---FREAV-----GTKEI---------ERTFDAE-----------------------------------ATVGDVLAAVEDEFP-------ELAGDILDDDG-------------------------DIQPHL--TVLRN-GRE-VVHLDGTATELSDG-DRLSIFPPV--------------------------AGG   93
222479963           LELRFFAT---FREAA-----GGKIV---------EAEFADG-----------------------------------SSVGDVLRELEAGYE-------GMNGR-LIVDG-------------------------GLAPQI--NVLKN-GRE-VLHLDGLDTSLDDG-DRLSVFPPV--------------------------AGG   95
134045266           LTIKFFAK---CREDF-----GEGLK---------ISIDLDE-----------------------------------VSILKLIEILENDYN-------LQLKEDF------------------------------ENGKI--IVSKD-FEI-----VSSNDVIDKN-SEIGIYPPV--------------------------SGG   84
150399418           LTVKFFAK---CREIH-----GNGLI---------ISTDLEK-----------------------------------LKISDLIEILSDNYN-------INLKEEF------------------------------KNGKI--IVSKD-FEI-----VSANDILTKD-SEIGIYPPV--------------------------SGG   84
45358920            LTIKFFAK---CREDF-----GEGLK---------ISIDLNE-----------------------------------VSVLELIEILEKDYN-------LQLKEDF------------------------------ENGRI--IVSKD-FEI-----VSSNDIINKN-SEIGIYPPV--------------------------SGG   84
154151667           IKVRFFAR---FRELL-----GTDII---------VTSQEG------------------------------------THLLTVVKKIAEKSQ-------DGYDAIFDAKG-------------------------DFREFV--IVMQN-GRR-VETATAASTPVTDG-DEIAVFPPV--------------------------AGG   92
116753334           IKIRAFAS---FREIL-----GKEMM---------VEIESG------------------------------------SNVSDLINHLCEIKP-------DLRRALYSDTG-------------------------ELRDYF--IIMLN-RRR-LNMPEDLGAALSDN-DEVAIFPPV--------------------------AGG   92
219851939           ITIRFFAQ---FRELL-----GERIQ---------LVVPEG------------------------------------TTITEAVQIVCRERN-------EAATALFGEDG-------------------------TFREYV--ILMQN-GIR-VNRTEAATTVVADE-DEVAVFPPV--------------------------AGG   93
18976915            VKVKYFAR---FRQLA-----GVDEE---------EIELPEG-----------------------------------ARVRDLIEEIKKRHE-------KFKEEVFGEGY-------------------------DEDADV--NIAVN-GRY-VSW----DEELKDG-DVVGVFPPV--------------------------SGG   90
242399827           VKVRYFAR---FRELS-----GTGEE---------IIELPKG-----------------------------------SKIRDLIEHIKSIHP-------DFKREAFSEGY-------------------------NDEVDI--NVSKN-GRY-ASF----DEELKEG-DIVALFPPT--------------------------SGG   92
33356787            VRVRYFAR---FRDLA-----GTSEE---------EIELKDG-----------------------------------ATIRDLIEEIKRRHE-------RFKSEVFGEDF-------------------------DEDADV--NVSLN-GRY-VSW----DEKLKDG-DVVGIFPPV--------------------------SGG   94
212224774           VKVRYFAR---FRSLV-----GTGEE---------ELEVPDG-----------------------------------IKVRELIDIIKERHP-------ILKNEVFAED--------------------------DDLADV--NVSRN-GRY-VSF----DEVLNDG-DTIALFPPV--------------------------SGG   88
240102133           VTVRYFAR---YRSLV-----GKGEE---------EVELPEG-----------------------------------ATVMDLIEKIKEMHP-------VLRNEVFAED--------------------------DDLADV--NVSRN-GRY-VRF----DETLREG-DVVAIFPPV--------------------------SGG   88
218883903           VKIRLFAS---LREKY-----GVSEI---------EVEVESDEFR-----------------------------EAIEKAGEILGRDFIDE--------------VLTSE-------------------------GYRSDR--ILLVN-GRH-IQFIE--ELKLRDG-DVIAIFPPI--------------------------AGG   91
119719092           VKFLLFAT---LRDKY-----GVPEV---------SVDCDGSLR------------------------------GCVEEAARILGRDFVEE--------------VFEGD-------------------------DYRRDR--IILVN-GRH-IQFVQ--STQLKDG-DVVAVFPPI--------------------------AGG  111
11499688            VRIEFYAT---LREKY------GKSI---------EVECDSTLR------------------------------GAFMAAAEKLGKDFLKE--------------VFDER------------------------GNFRDDR--IITVN-GRN-IKDEM--IEKLSEN-ARIAVFPPV--------------------------AGG   89
124485016           ITVKAFAT---FREVM------DMKV---------ELEFPAG-----------------------------------ATIKTLLSELTARYM-------GLDALLFAAPG-------------------------TLRDFV--NILKN-GRN-IHFIAGLDTPLDEG-DMIALFPPA--------------------------AGG   92
219852168           VTVRGYAD---LRLVI-----GDQ-----------EVVLPEV-----------------------------------ATVQHLLDLLIVDHP-------VISTALFDQAG-------------------------ALLPAV--NILKN-GRN-IFFLNGLSTLLEEG-DLISVFPPV--------------------------AGG   91
13541625            VTVRYYAN---LRSVT-----GKKLE---------EFDGI-------------------------------------KNIDDLISLLNQEYG-------EKFRKLMYNGN-------------------------ELYPNV--IILLN-GNN-INSIEGLKTPLKDG-DNIDVFPPV--------------------------AGG   91
16081944            VTVRYYAT---LRPIT-----KKKEE---------TFNGI-------------------------------------SKISELLERLKVEYG-------SEFTKQMYDGN-------------------------NLFKNV--IILVN-GNN-ITSMKGLDTEIKDD-DKIDLFPPV--------------------------AGG   90
2g1e_chainA_p001    VTVRYYAT---LRPIT-----KKKEE---------TFNGI-------------------------------------SKISELLERLKVEYG-------SEFTKQMYDGN-------------------------NLFKNV--IILVN-GNN-ITSMKGLDTEIKDD-DKIDLFPPV--------------------------AGG   90
124028367           LHVLGFREGNPFHHVL---------------------ELSEE----------------------------------CVTIECIIERVGVEGAI-----YALGRRVE-------------------------------------------------------DKLPEGASEVYLLSPPPGPVAARLLGPNERLDFNMVYEVLSLRGG   95
242399599           LTLTYGGP---FYEKT-----KKHKE---------IIELDKN----------------------------------SITVQELVKFLFEQYP-------ELAQFFENNPE-------------------------KIFEFS--TIIVR-GRV-LIPQDLPRVHLSDG-EEVFFLPVV--------------------------HGG   96
124026993           VTVVPGGK-------------TR------------ILE-------------------------------------------------FGAMP-------RVRDILEKL---------------------------GIPLEA--AVVVR-TRD--VKPLLPDEHVGDG-EDVTVYVAA--------------------------STG   71
124027961           VRVRLVSL---LREAV-----GGSEV---------VIEVGED-----------------------------------ATLRDVLKTLFTRYP-------GLEKLVRELE--------------------------ARGLDI--VYLVN-GRF-----SGLDQQVRDG-DEIVILPPA--------------------------SGG   89
219851847           ITLELPDL---TRKLC--------------------SPSGD----------------------------------------------------------TIEELLVAAG--------------------------VNPAEV--IVVRD-GVL-----LPEDTPLSDG-DHLRVISIV--------------------------HGG   65
15921923            VTVQLVRE--------------NKEI---------VVELPEK-----------------------------------ATVKDLLKKIGYR-----------------------------------------------VQGS--VVVKD-GLP-----IVEDERLNDG-DKVQVFLAA--------------------------SGG   68
70607385            VTVELVRE--------------NKII---------EVELPER-----------------------------------ARVRDLLKKIGYR-----------------------------------------------VQGS--VVVKN-SLP-----IIEDEELKDG-DKLRVFLAA--------------------------SGG   68
146304182           VTVYLPRE--------------KREK---------EVELNPN-----------------------------------STVRDLVRKLGLT-----------------------------------------------VQGS--VVLRD-GVP-----LLEDERIKEG-EKLTVVQTA--------------------------SGG   68
218884697           ---------------------EQAPR---------ILDVGE------------------------------------ISVSELLRRLGLS-----------------------------------------------KSEH--IVLRE-GIP-----LTEDDIIRDG-DEVVVYLVK--------------------------SGG   61
212223899           IRIRLMGV---FAYLA-----KAREL---------NVRIEDK-----------------------------------KTVDEILREVIPRYD--------------------------------------------EFKEK--IIFIN-GQP-----ARGDAEVTDG-DEIKVMPVL--------------------------SGG   76
242398470           VKIRLMGA---FAHLA-----KAREL---------EVKLEGP-----------------------------------KTANEILREVIPRYD--------------------------------------------EFHDK--IIFIN-GKP-----AKGEAIVEEN-DEIKVMPVL--------------------------SGG   76
18977977            MKIKLMGV---FAHLA-----GAEEV---------EVRIAGK-----------------------------------RQVGEVLREVIPRFD--------------------------------------------EIKEK--IIIIN-GKI-----AREDAEVSDN-DVVKIMPVP--------------------------SGG   79
33356700            MRIRLMGI---FSHLA-----GSQEV---------EIQINGK-----------------------------------RKVGEVLREIIPRFD--------------------------------------------EVKEK--IIIIN-EKV-----ATEDAEVTNE-DFVKVMPVL--------------------------SGG   78
33356745            IKVKVIGR--------------GIEK---------EIEWREG-----------------------------------MKVKDVLRAVGFNT-----------------------------------------------ESA--IAKVN-GKV-----ALEDKEVKDG-DFVEVIPVV--------------------------SGG   69
242399406           IKIKVIGR--------------KIEK---------ELEYQKG-----------------------------------MKVKDILKAVGFNT-----------------------------------------------ESA--IAKVN-GKV-----ALEDDPIKDN-DYVEVIPVV--------------------------SGG   75
33359535            IKVKVIGR--------------NIEK---------EIEWREG-----------------------------------MKVRDILRAVGFNT-----------------------------------------------ESA--IAKVN-GKV-----VLEDDEVKDG-DFVEVIPVV--------------------------SGG   71
212223374           IRVKVLGR--------------GIEK---------ELEWRKG-----------------------------------MKIADVLREVGFNT-----------------------------------------------ESA--IAKLN-GRV-----ALEDENVKDG-DYVEVIPVV--------------------------SGG   67
240102862           IRVKVLGR--------------GIEK---------EVEWKRG-----------------------------------MKVADVLREVGFNT-----------------------------------------------ESA--IARVN-GKV-----VLEDDPVGDG-DYVEVIPVV--------------------------SGG   70
18977854            VKVKLYGE---LALKH------GVEV---------EVEVKEG-----------------------------------ARVVDILRMLKISP-----------------------------------------------SEH--HLILN-ERK-----VSLDHQLKDG-DVLKILPVV--------------------------YGG   73
242399115           IKVRLYGE---LALKH------GAEV---------EIEIEKE-----------------------------------ATVGDILKLLRISE-----------------------------------------------SEH--HLILN-EKK-----VSKNHPLKEG-DTLKVLSVV--------------------------YGG   73
212224959           VILILYGE---VALRF------SPRM---------ELELEER-----------------------------------TTVGELLRELGISA-----------------------------------------------VEH--HILVN-ERK-----VDHSRVLKDG-DVVKVLPVI--------------------------YGG   73
150400275           IFAKIGE----------------NSF---------ELEFSKD-----------------------------------ITINDILNLLKI-------------------------------------------------KDV--LVVRN-GEI-----LSDDDLIYEN-ETLRLVPIV--------------------------SGG   64
134045956           VFIKNAG----------------ELS---------EIELPEN-----------------------------------AKLKDFIEKMGI-------------------------------------------------KDN--LVVRN-GEI-----LSETDDLSEN-DTLRLVPIV--------------------------SGG   64
45358218            VFIKNAG----------------ELS---------EIELPEN-----------------------------------AKLKDFIEKMDI-------------------------------------------------KDN--LVVRN-GEI-----LSETDDLSEN-DTLRLVPIV--------------------------SGG   64
118431725           IKVRLVPE--------------GGER---------VIQAERP-----------------------------------LKAGELLRMLGMNE-----------------------------------------------EVA--VVLRD-GKP-----LLPEDLVSPG-ETVDVVRVL--------------------------SGG   74
126465688           VRVVFLAR---AGDVV-----GKHFH---------DFILDDN-----------------------------------ATLNDLIEAIGKELS------PRFYRGVKN-----------------------------GRLVF--AIFVN-GKP----VDEPNYKLKDN-DRVVFTTPE--------------------------MGG   89
218884045           VKVVFIGI---IAEAI-----GTHIE---------EYSIQEN-----------------------------------TRLNELLDVIAEKHP-------FLQEILRN------------------------------IPLI--NTYVN-GRH-----VDLNHVLEDN-DEVIIAPPF-------------------------FEGG   86
16082411            AMIKVRGH---------------IRK---------ELTIESE-----------------------------------RKVSDLMKDLGLN-----------------------------------------------EDEY--VVIVN-GSP-----VLGDHIVKKE-DDVTILEVF--------------------------SGG   67
13540947            NMITVKGH---------------IKK---------SVEIDRE-----------------------------------MSVGDILKDLGLP-----------------------------------------------EEEY--VVIVN-GKP-----VLADHTVKKD-DDVVILEVF--------------------------SGG   68
48477609            -MIRIKGS---------------ENK---------TIEMDHE-----------------------------------LTINDIIKQYKID-----------------------------------------------AERY--AIILN-GSP-----ATEDENVRPE-DDLLFLEVF--------------------------SGG   64
13541400            VRVRYFAY---FKEKV-----GKDEE---------YFELSPG-----------------------------------SNVGTLINEVMKKYK-------NIFN----------------------------------PKDT--LIAVN-YKY-----VDQNYNIAEG-DTVAIMPHV--------------------------SGG   90
16082053            VTVKYFAY---YRERV-----GKDSE---------KIQLDQG-----------------------------------KRISDLIDMLMARYP-------GIFN----------------------------------EKNL--FVARN-YRY-----ADRGEALSDG-DVVAIMPHV--------------------------SGG   85
16081733            IRIRVLSH---FSSAL-----GFDES---------SFSIGEG-----------------------------------ITFAELRSKLIEQFS-------AFVR----------------------------------PIEI--GMYVN-MKP-----VPDDYTMMDG-DIVYVFPRS--------------------------STP   80
Consensus_aa:       lplph.t....h.p.h....................hph.......................................ph.phlp.l.....................................................h...lh.s.tp.......s.p..l.cs.D.l.lh.sh...........................GG
Consensus_ss:       eeeee      hhhh         ee         eee                                         hhhhhhhhhhh         hhhhhh                                  ee  eeee                      eeeee
```

The alignment for all β-grasp fold domains containing proteins from archaeal genomes was constructed by PROMALS-3D program with two reference structures (2g1e and 3dwm). Sequences are denoted by their GI numbers were retrieved from arCOG database (ftp://ftp.ncbi.nih.gov/pub/wolf/COGs/arCOG/). The consensus predicted by PSIPRED program secondary structures are shown in the last line. If the fraction of helix or strand predictions among representative sequences in a position is larger than 0.5, the consensus letter is "h" or "e", respectively. **Conserved** amino acid residues (“Consensus_aa” line) - uppercase letters (such as **G**); **aliphatic** residues (**I, V, L**): l; **aromatic** residues (**Y, H, W, F**): @; **hydrophobic** residues (**W, F, Y, M, L, I, V, A, C, T, H**): h; **alcohol** residues (**S, T**): o; **polar** residues (**D, E, H, K, N, Q, R, S, T**): p; **tiny** residues (**A, G, C, S**): t; **small** residues (**A, G, C, S, V, N, D, T, P**): s; **bulky** residues (**E, F, I, K, L, M, Q, R, W, Y**): b; **positively charged** residues (**K, R, H**): +; **negatively charged** residues (**D, E**): -; **charged** (**D, E, K, R, H**): c

```
URM1
72387818_Trypanosoma brucei         MSN-HNHITVQFAGGCELLFAKQTSLQLDGVVP--TGTNLNGLVQLLKTNYVKERP-DLLVDQT-------------------------GQTLRPGILVLVNSCDAEVVGGMDYVLNDGDTVEFISTLHGG  102
154336581_Leishmania braziliensis   MDVMHKKIKISLSGGCELLFNKEESITLADVVP--VGATVAELIDILRRDYIKERP-ELFVDAT-------------------------GTNVRPGILVLVNGCDAEVFGGVQHVLEDGDEVEFVSTLHGG  103
159489488_Chlamydomonas reinhardtii M----VKVKIEFSGGLELLFGNQKQHDVDVPVQEGKQLTAGHLIAWTRDNMLRERP-EL--FVK-------------------------GHTVRPGILVLINECDWELSGATESTISDGDVVVFISTLHGG   99
13569870_Homo sapiens               MAA-PLSVEVEFGGGAELLFDGIKKHRVTLPGQ-EEPWDIRNLLIWIKKNLLKERP-EL--FIQ-------------------------GDSVRPGILVLINDADWELLGELDYQLQDQDSVLFISTLHGG  101
66810572_Dictyostelium discoideum   -----MKVKIELSGGLELLFDKKKVHEIEFSDK--NEIPLKDLILYMRDNLLKERS-EL--FVV-------------------------DDTVRPGILVLINDADWELFGGISYNVEDKDTIIFISTLHGG   96
295442748_Schizosaccharomyces pombe -----MAIKVELLGGLDLLFNKQKALSLSLSNL--GSTKLGSLIDYMAQIIEKPSQKDL--FIL-------------------------NGTVRPGIIVLVNDQDWELLEKEEYNLEEGDEVVFVSTLHGG   97
124804635_Plasmodium falciparum     MK---TKVELKFLGGLESYLANKSKNYVSLEIE-SEEFNFENLIAYIRNHIIVDRK-DVFSDFVMSDGNVKSCNVMIDDKEYSNYNLSDKGKIKPGIIVLINEYDWEILDTYTYKIKNNDKICFLSTLHGG  126
6322183_Saccharomyces cerevisiae    M----VNVKVEFLGGLDAIFGKQRVHKIKMDKE--DPVTVGDLIDHIVSTMINNPN-DVSIFIE-------------------------DDSIRPGIITLINDTDWELEGEKDYILEDGDIISFTSTLHGG   99

arCOG00540
70606476___Sulac__arCOG00540        -----MSVKIRLKGPLATRLGR-DEFVISLKAD--------NLLDILKEL--DKEE-KL--LIN-------------------------GNKIRSGYILLINGIDYRLLNG---KLKDGDVVDILPINHGG   84
146305007__Metse__arCOG00540        ------MVKVVLRGPLVSIFSG-NEFSVKGD----------NLISILSKI--DKRG-I---IVS-------------------------DGRIKPGYLILVNGMDFRLLKK--EVLEDSDTVDIIPINHGG   81
15897228___Sulso__arCOG00540        ------MPKVILKGPLISQFNF-REIYVNDR----------ELLRVLVKI--DSKK-HLI-LNE-------------------------SNQLKSGILILINGKDWRLYRN--QLLNDNDIIEIIPINHGG   83
Consensus_aa:                       ........cl.h.Gsh...hs..p...lp............pLl.hh.p....pp..cl................................pl+sGhllLlN..Dhcl.......lps.D.l.hlsh.HGG
Consensus_ss:                            eeeeeee          eeeeeeee         hhhhhhhhhhh       e   ee                            ee  eeeeee    eeee   eee     eeeee
```

The alignment was constructed by PROMALS-3D program. Sequences are denoted by their GI number and full species name for URM1 family and GI number and abbreviated species names for arCOG00540 family. Amino acids within alignment are colored according to PSIPRED secondary structure prediction - red: alpha-helix, blue: beta-strand. The consensus predicted secondary structures are shown in the last line in each block. If the fraction of helix or strand predictions among representative sequences in a position is larger than 0.5, the consensus letter is "h" or "e", respectively. **Conserved** amino acid residues: bold and uppercase letters (such as **G**); **aliphatic** residues (**I, V, L**): l; **aromatic** residues (**Y, H, W, F**): @; **hydrophobic** residues (**W, F, Y, M, L, I, V, A, C, T, H**): h; **alcohol** residues (**S, T**): o; **polar** residues (**D, E, H, K, N, Q, R, S, T**): p; **tiny** residues (**A, G, C, S**): t; **small** residues (**A, G, C, S, V, N, D, T, P**): s; **bulky** residues (**E, F, I, K, L, M, Q, R, W, Y**): b; **positively charged** residues (**K, R, H**): +; **negatively charged** residues (**D, E**): -; **charged** (**D, E, K, R, H**): c

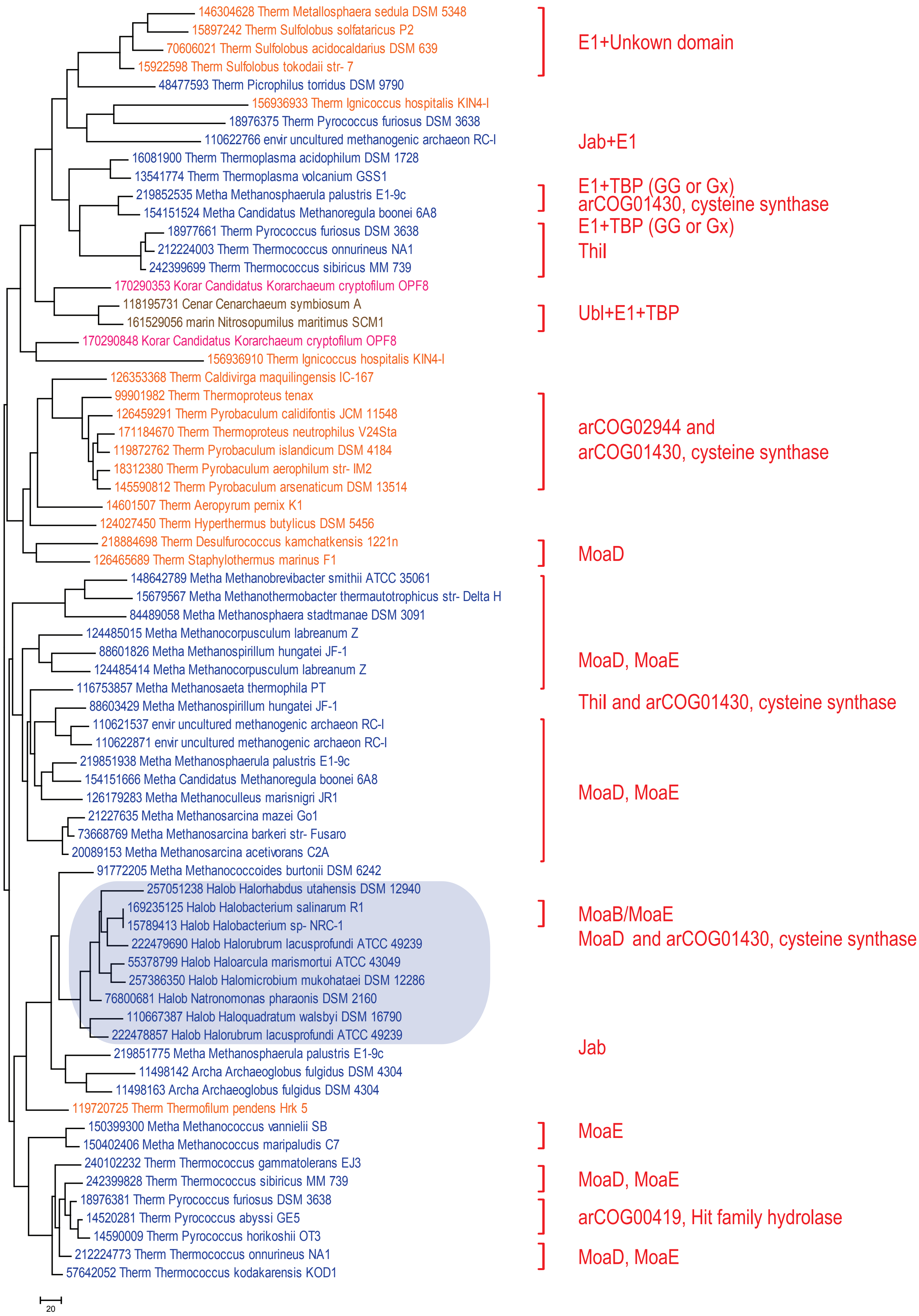

146304628 Therm Metallosphaera sedula DSM 5348
15897242 Therm Sulfolobus solfataricus P2
70606021 Therm Sulfolobus acidocaldarius DSM 639
15922598 Therm Sulfolobus tokodaii str- 7
48477593 Therm Picrophilus torridus DSM 9790
156936933 Therm Ignicoccus hospitalis KIN4-I
18976375 Therm Pyrococcus furiosus DSM 3638
110622766 envir uncultured methanogenic archaeon RC-I
16081900 Therm Thermoplasma acidophilum DSM 1728
13541774 Therm Thermoplasma volcanium GSS1
219852535 Metha Methanosphaerula palustris E1-9c
154151524 Metha Candidatus Methanoregula boonei 6A8
18977661 Therm Pyrococcus furiosus DSM 3638
212224003 Therm Thermococcus onnurineus NA1
242399699 Therm Thermococcus sibiricus MM 739
170290353 Korar Candidatus Korarchaeum cryptofilum OPF8
118195731 Cenar Cenarchaeum symbiosum A
161529056 marin Nitrosopumilus maritimus SCM1
170290848 Korar Candidatus Korarchaeum cryptofilum OPF8
156936910 Therm Ignicoccus hospitalis KIN4-I
126353368 Therm Caldivirga maquilingensis IC-167
99901982 Therm Thermoproteus tenax
126459291 Therm Pyrobaculum calidifontis JCM 11548
171184670 Therm Thermoproteus neutrophilus V24Sta
119872762 Therm Pyrobaculum islandicum DSM 4184
18312380 Therm Pyrobaculum aerophilum str- IM2
145590812 Therm Pyrobaculum arsenaticum DSM 13514
14601507 Therm Aeropyrum pernix K1
124027450 Therm Hyperthermus butylicus DSM 5456
218884698 Therm Desulfurococcus kamchatkensis 1221n
126465689 Therm Staphylothermus marinus F1
148642789 Metha Methanobrevibacter smithii ATCC 35061
15679567 Metha Methanothermobacter thermautotrophicus str- Delta H
84489058 Metha Methanosphaera stadtmanae DSM 3091
124485015 Metha Methanocorpusculum labreanum Z
88601826 Metha Methanospirillum hungatei JF-1
124485414 Metha Methanocorpusculum labreanum Z
116753857 Metha Methanosaeta thermophila PT
88603429 Metha Methanospirillum hungatei JF-1
110621537 envir uncultured methanogenic archaeon RC-I
110622871 envir uncultured methanogenic archaeon RC-I
219851938 Metha Methanosphaerula palustris E1-9c
154151666 Metha Candidatus Methanoregula boonei 6A8
126179283 Metha Methanoculleus marisnigri JR1
21227635 Metha Methanosarcina mazei Go1
73668769 Metha Methanosarcina barkeri str- Fusaro
20089153 Metha Methanosarcina acetivorans C2A
91772205 Metha Methanococcoides burtonii DSM 6242
257051238 Halob Halorhabdus utahensis DSM 12940
169235125 Halob Halobacterium salinarum R1
15789413 Halob Halobacterium sp- NRC-1
222479690 Halob Halorubrum lacusprofundi ATCC 49239
55378799 Halob Haloarcula marismortui ATCC 43049
257386350 Halob Halomicrobium mukohataei DSM 12286
76800681 Halob Natronomonas pharaonis DSM 2160
110667387 Halob Haloquadratum walsbyi DSM 16790
222478857 Halob Halorubrum lacusprofundi ATCC 49239
219851775 Metha Methanosphaerula palustris E1-9c
11498142 Archa Archaeoglobus fulgidus DSM 4304
11498163 Archa Archaeoglobus fulgidus DSM 4304
119720725 Therm Thermofilum pendens Hrk 5
150399300 Metha Methanococcus vannielii SB
150402406 Metha Methanococcus maripaludis C7
240102232 Therm Thermococcus gammatolerans EJ3
242399828 Therm Thermococcus sibiricus MM 739
18976381 Therm Pyrococcus furiosus DSM 3638
14520281 Therm Pyrococcus abyssi GE5
14590009 Therm Pyrococcus horikoshii OT3
212224773 Therm Thermococcus onnurineus NA1
57642052 Therm Thermococcus kodakarensis KOD1
20
E1+Unkown domain
Jab+E1
E1+TBP (GG or Gx)
arCOG01430, cysteine synthase
E1+TBP (GG or Gx)
ThiI
Ubl+E1+TBP
arCOG02944 and
arCOG01430, cysteine synthase
MoaD
MoaD, MoaE
ThiI and arCOG01430, cysteine synthase
MoaD, MoaE
MoaB/MoaE
MoaD and arCOG01430, cysteine synthase
Jab
MoaE
MoaD, MoaE
arCOG00419, Hit family hydrolase
MoaD, MoaE

Supplementary Figure S3. Phylogenetic analysis of archaeal E1-like proteins (arCOG001676).

The maximum likelihood tree was reconstructed by MOLPHY program (Adachi and Hasegawa 1992); 245 informative positions were used for the reconstruction. Color codes, sequence identifiers and species abbreviations are the same as in the Figure 1. Fusions are shown by the sequence of domains delimited by “+” symbol. Genes and domains associated with E1-like enzyme are the following: MoaE - Molybdopterin converting factor, large subunit; MoaD – Ubl protein of MoaD subfamily; ThiI - Thiamine biosynthesis ATP pyrophosphatase; MoaB - Molybdopterin biosynthesis enzyme; TBP - TATA-binding protein (TBP)-like fold.
